# Supplementary material for: Resolving the full spectrum of human genome variation using Linked-Reads
Source: Genome Res. 2019 Apr;29(4):635–45. doi: 10.1101/gr.234443.118 (PMC6442396; doi:10.1101/gr.234443.118)
Supplement: Supplemental Material [file supp_gr.234443.118_Supplemental_longranger-2.2.zip › longranger-2.2.tar.bz2!/lr2.2-public/lib/stan/cmdstan/stan/doc/api/html/expression__grammar_8hpp_source.html]

Stan: src/stan/gm/grammars/expression\_grammar.hpp Source File


|  |  |
| --- | --- |
|  | Stan  2.5.0  probability, sampling & optimization |


- Main Page
- Related Pages
- Namespaces
- Classes
- Files

- File List
- File Members

All Classes Namespaces Files Functions Variables Typedefs Enumerations Enumerator Friends Macros Pages

- src
- stan
- gm
- grammars

expression\_grammar.hpp

Go to the documentation of this file.

1 #ifndef STAN\_\_GM\_\_PARSER\_\_EXPRESSION\_GRAMMAR\_\_HPP

2 #define STAN\_\_GM\_\_PARSER\_\_EXPRESSION\_GRAMMAR\_\_HPP

3

4 #include <string>

5 #include <sstream>

6 #include <vector>

7

8 #include <boost/spirit/include/qi.hpp>

9

10 #include <stan/gm/ast.hpp>

11 #include <stan/gm/grammars/expression07\_grammar.hpp>

12 #include <stan/gm/grammars/term\_grammar.hpp>

13 #include <stan/gm/grammars/whitespace\_grammar.hpp>

14

15 namespace stan {

16

17  namespace gm {

18

19  template <typename Iterator>

20  struct term\_grammar;

21

22  template <typename Iterator>

23  struct expression\_grammar;

24

25  template <typename Iterator>

26  struct expression07\_grammar;

27

28  template <typename Iterator>

29  struct expression\_grammar

30  : public boost::spirit::qi::grammar<Iterator,

31  expression(var\_origin),

32  whitespace\_grammar<Iterator> > {

33

34  expression\_grammar(variable\_map& var\_map,

35  std::stringstream& error\_msgs);

36

37  variable\_map& var\_map\_;

38

39  std::stringstream& error\_msgs\_;

40

41  expression07\_grammar<Iterator> expression07\_g;

42

43

44  boost::spirit::qi::rule<Iterator,

45  expression(var\_origin),

46  whitespace\_grammar<Iterator> >

47  expression\_r;

48

49  boost::spirit::qi::rule<Iterator,

50  expression(var\_origin),

51  whitespace\_grammar<Iterator> >

52  expression09\_r;

53

54  boost::spirit::qi::rule<Iterator,

55  expression(var\_origin),

56  whitespace\_grammar<Iterator> >

57  expression10\_r;

58

59  boost::spirit::qi::rule<Iterator,

60  expression(var\_origin),

61  whitespace\_grammar<Iterator> >

62  expression14\_r;

63

64

65  };

66

67  }

68 }

69

70 #endif

expression07\_grammar.hpp

stan::gm::expression\_grammar::expression09\_r

boost::spirit::qi::rule< Iterator, expression(var\_origin), whitespace\_grammar< Iterator > > expression09\_r

**Definition:** expression\_grammar.hpp:52

stan::gm::expression\_grammar::expression07\_g

expression07\_grammar< Iterator > expression07\_g

**Definition:** expression\_grammar.hpp:41

stan::gm::expression\_grammar::expression14\_r

boost::spirit::qi::rule< Iterator, expression(var\_origin), whitespace\_grammar< Iterator > > expression14\_r

**Definition:** expression\_grammar.hpp:62

stan::gm::variable\_map

**Definition:** ast.hpp:439

stan::gm::expression\_grammar::var\_map\_

variable\_map & var\_map\_

**Definition:** expression\_grammar.hpp:37

stan::gm::expression

**Definition:** ast.hpp:216

term\_grammar.hpp

stan::gm::expression\_grammar::error\_msgs\_

std::stringstream & error\_msgs\_

**Definition:** expression\_grammar.hpp:39

ast.hpp

stan::gm::whitespace\_grammar

**Definition:** whitespace\_grammar.hpp:11

whitespace\_grammar.hpp

stan::gm::expression\_grammar::expression\_r

boost::spirit::qi::rule< Iterator, expression(var\_origin), whitespace\_grammar< Iterator > > expression\_r

**Definition:** expression\_grammar.hpp:47

stan::gm::expression\_grammar::expression10\_r

boost::spirit::qi::rule< Iterator, expression(var\_origin), whitespace\_grammar< Iterator > > expression10\_r

**Definition:** expression\_grammar.hpp:57

stan::gm::expression\_grammar::expression\_grammar

expression\_grammar(variable\_map &var\_map, std::stringstream &error\_msgs)

**Definition:** expression\_grammar\_def.hpp:106

stan::gm::expression07\_grammar

**Definition:** expression07\_grammar.hpp:26

stan::gm::var\_origin

int var\_origin

**Definition:** ast.hpp:411


---

[ Stan Home Page ]

*© 2011–2014,
Stan Development Team.*
